# Stemness, Pluripotentiality, and Wnt Antagonism: sFRP4, a Wnt antagonist Mediates Pluripotency and Stemness in Glioblastoma

**DOI:** 10.3390/cancers11010025

**Published:** 2018-12-27

**Authors:** Gurubharathi Bhuvanalakshmi, Naisarg Gamit, Manasi Patil, Frank Arfuso, Gautam Sethi, Arun Dharmarajan, Alan Prem Kumar, Sudha Warrier

**Affiliations:** 1 Division of Cancer Stem Cells and Cardiovascular and Neuronal Regeneration, School of Regenerative Medicine, Manipal Academy of Higher Education (MAHE), Bangalore 560 065, India; bhuvanabharathi@gmail.com (G.B.); 12naisarg@gmail.com (N.G.); mansipatil01@gmail.com (M.P.); 2 Stem Cell and Cancer Biology Laboratory, School of Pharmacy and Biomedical Sciences, Curtin Health Innovation Research Institute, Curtin University, Perth, WA 6102, Australia; frank.arfuso@curtin.edu.au (F.A.); a.dharmarajan@curtin.edu.au (A.D.); 3 School of Human Sciences, The University of Western Australia, Perth, WA 6009, Australia; 4 Department of Pharmacology, Yong Loo Lin School of Medicine, National University of Singapore, Singapore 117600, Singapore; phcgs@nus.edu.sg; 5 Cancer Science Institute of Singapore, National University of Singapore, Singapore 117599, Singapore; 6 Cancer Program, Medical Science Cluster, Yong Loo Lin School of Medicine, National University of Singapore, Singapore 119228, Singapore; 7 Curtin Medical School, Curtin University, Perth, WA 6102, Australia; 8 Cuor Stem Cellutions Pvt Ltd., School of Regenerative Medicine, Manipal Academy of Higher Education (MAHE), Bangalore 560 065, India

**Keywords:** Wnt, sFRP4, glioblastoma, pluripotency, p53

## Abstract

Background: Chemotherapeutic resistance of glioblastoma has been attributed to a self-renewing subpopulation, the glioma stem cells (GSCs), which is known to be maintained by the Wnt β−catenin pathway. Our previous findings demonstrated that exogeneous addition of the Wnt antagonist, *secreted fizzled-related protein 4* (*sFRP4*) hampered stem cell properties in GSCs. Methods: To understand the molecular mechanism of *sFRP4*, we overexpressed *sFRP4* (sFRP4 OE) in three human glioblastoma cell lines U87MG, U138MG, and U373MG. We also performed chromatin immunoprecipitation (ChIP) sequencing of sFRP4 OE and RNA sequencing of sFRP4 OE and sFRP4 knocked down U87 cells. Results: We observed nuclear localization of sFRP4, suggesting an unknown nuclear role. ChIP-sequencing of sFRP4 pulldown DNA revealed a homeobox *Cphx1*, related to the senescence regulator *ETS proto-oncogene 2* (*ETS2*). Furthermore, *miRNA885*, a p53-mediated apoptosis inducer, was upregulated in sFRP4 OE cells. RNA sequencing analysis suggested that sFRP4-mediated apoptosis is via the Fas-p53 pathway by activating the Wnt calcium and reactive oxygen species pathways. Interestingly, sFRP4 OE cells had decreased stemness, but when knocked down in multipotent mesenchymal stem cells, pluripotentiality was induced and the Wnt β-catenin pathway was upregulated. Conclusions: This study unveils a novel nuclear role for *sFRP4* to promote apoptosis by a possible activation of DNA damage machinery in glioblastoma.

## 1. Introduction

Glioblastoma multiforme (GBM) is one of the most aggressive and devastating types of brain tumor in adults. Although the standard mode of treatment is surgical resection, followed by radiation and chemotherapy with temozolomide, the prognosis remains poor with a median survival range of 12–15 months. The properties of GBM such as high proliferation, resistance to chemo and radiotherapy, and infiltrative nature are the primary reasons for its highly malignant and aggressive nature. These properties are the inherent and defining feature of cancer stem cells (CSCs), and CSCs from glioblastoma were first identified in 2003 by Singh et al. [1]. This population of cells, by virtue of their differential regulation, are able to self-renew, aberrantly differentiate, initiate tumorigenesis, are resistant to drugs, have active DNA repair capacity, express ABC transporters, are capable of de novo tumor formation when implanted to xenograft models, and express *CD133* and *nestin*, which gave them the capacity to form neurospheres [2,3]. One of the major challenges in GBM treatment remains tumor recurrence, which is initiated by the drug resistant glioma stem cells.

The unique properties of CSCs are determined by their aberrant cellular signaling mechanisms. Specifically, dysregulation of the key developmental pathways, namely Wnt, Notch, and Sonic hedgehog pathways, has been implicated in modulation of CSCs. Aberrant activation of Wnt β-catenin was observed in multiple tumor types and correlated with increased proliferative, migrative, and invasive properties, and hence has been identified as a hallmark signaling pathway of cancers. The Wnt β-catenin pathway has been found to promote symmetric cell division self-renewal in prostate CSCs [4]. Additionally, in breast CSCs, Wnt β-catenin mediators increased the migratory and metastatic potential [5]. Wnt β-catenin signaling plays an important role in the maintenance of chemoresistance in cancer cells by modulating the transcription of multidrug resistance genes *ABCB1/MDR-1*, which are also expressed in normal somatic stem cells [6].

Therefore, targeting CSCs by employing inhibitors of the Wnt pathways would help in effective destruction of the tumor-initiating cell types, which would further mitigate recurrence and relapse, a malevolent after-effect of currently used chemotherapeutics. Pathway-specific antagonism using molecular target agents such as Wnt antagonist proteins will help in targeting CSCs whilst causing no damage to normal tissue cells. Among the Wnt antagonists, the secreted frizzled-related protein (sFRP) family of sFRP1-5 inhibitors has a far reaching effect across the Wnt pathways, being able to bind both the Wnt ligand and frizzled receptor. Silencing of *sFRP* genes via hypermethylation at the promoter region has been reported in hepatocarcinoma [7,8] and glioblastoma [9]. Previously, we have reported the ability of sFRP4 to chemosensitize CSCs from glioma and head and neck cancers and improve the response to drugs [10,11,12]. In breast CSCs, *sFRP4* overexpression resulted in improved response to drugs and decreased CSC population and stemness [13]. Although this Wnt antagonist clearly has an ability to target and kill CSCs, its specific mechanism of action in inducing apoptosis and targeting the stem-like phenotype has not yet been elucidated.

In this study we focus on the molecular mechanisms of *sFRP4* in inducing apoptosis. Transcriptome analysis revealed that *sFRP4* promotes apoptosis by possible activation of p53-mediated Fas-FasL cascade, Wnt calcium pathway, and senescence in addition to the conventional inhibition of the Wnt β-catenin pathway. Interestingly, *sFRP4* also has a possible direct role in regulating stemness and pluripotentiality.

## 2. Materials and Methods

### 2.1. Cell Culture

U87MG, U373MG, and U138MG glioblastoma cell lines were obtained from NCCS, Pune and were cultured and maintained as described earlier [11]. Human Wharton’s jelly mesenchymal stem cells (WJMSCs) were obtained from human placenta after approval from the Institutional Ethical Committee (IEC) of Manipal Hospital, Bangalore, India, and isolated and cultured as described previously [14]. The human embryonic stem cell (hESC) line, HUES-7 was obtained as a generous gift from Harvard University Stem Cell Institute (Prof Douglas Melton) and were cultured in embryonic stem cell (ESC) medium on mitomycin-C inactivated mouse embryonic fibroblast (MEF)-coated dishes at 37 °C in a 5% CO_2_ incubator.

### 2.2. sFRP4 Overexpression

U87MG, U373MG, and U138MG cells were transfected with 1 µg/µL of pEGFP N1 plasmid (Clontech, Palo Alto, CA, USA) with the sFRP4 gene insert mediated by Lipofectamine 3000 (Invitrogen, Carlsbad, CA, USA) in Opti-MEM reduced serum medium for 24–48 h. The transfection rate was confirmed by green fluorescent protein (GFP) expression under an Eclipse TE2000-U fluorescence microscope (Nikon, Tokyo, Japan) equipped with Qimaging- QICAM-fast 1394 (Surrey, BC, Canada) to determine sFRP4 gene expression [13]. 

### 2.3. sFRP4 siRNA Silencing

Upon reaching ~80% confluency, U87MG, U373MG, and U138MG cells were transfected with 2 nM sFRP4 SiRNA (Qiagen-Xeragon, Germantown, MD, USA) by using Lipofectamine 3000 (Invitrogen) with Opti-MEM reduced serum medium for 24 h without antibiotic supplement. sFRP4-SiRNA efficiency was assessed by gene expression analysis. The primer details are provided in the Table S1.

### 2.4. Cell Viability, Proliferation, Reactive Oxygen Species (ROS), and Caspase Assays

The cell viability, proliferation, reactive oxygen species (ROS), and caspase assays were performed on U87, U373, and U138 cells after sFRP4 overexpression and silencing as previously described [12]. 

### 2.5. Secondary Sphere Forming Assay

To observe the secondary sphere forming ability of the cells after treatment, the spheroids were quantified using anchorage-independent culturing on soft agar as described previously [13].

### 2.6. Chromatin Immunoprecipitation (ChIP) Sequencing

sFRP4 overexpressing (sFRP4 OE) U87 cells were used for the ChIP pull-down against rabbit sFRP4 monoclonal antibody (Abcam, Cambridge, MA, USA) along with normal U87 control. sFRP4 OE pull-down DNA samples were taken for whole-genome ChIP sequencing with input DNA control from U87 cells using Illumina HiSeq 2500 System (Illumina Inc., San Diego, CA, USA).

### 2.7. MicroRNA 885 Analysis

Total RNA was isolated from U87 treated cells by using the Trizol method. miRCURY LNA^TM^ Universal RT microRNA PCR kit (Exiqon, Woburn, MA, USA), hsa-miR-885-5p (Accession ID: MIMAT0004947; Sequence-UCCAUUACACUACCCUGCCUCU) and hsa-miR-885-3p (Accession ID: MIMAT0004948; Sequence-AGGCAGCGGGGUGUAGUGGAUA) were used in the miRNA885 analysis. miRCURY LNA miRNA Detection probe (hsa-miR-885-3p; Exiqon) was used to localize mature miRNA activity in treated U87 cells by using in situ hybridization as previously described [15].

### 2.8. RNA Sequencing

Total RNA from U87 cells subjected to either sFRP4 OE or sFRP4 silencing and untreated cells was isolated and sequenced with Illumina HiSeq 2500 System (Illumina Inc.) for the whole transcriptome analysis.

### 2.9. Bioinformatics Analysis

DNA binding prediction: sFRP4-specific DNA binding competence was identified using a DNA Binding Protein prediction software, DNABIND (http://dnabind.szialab.org/) [16]. 

Mapping and Binding Site Prediction of ChIP-Seq Data: ChIP sequencing from sFRP4 OE pull-down DNA and input DNA results were mapped using Burrows-Wheeler Aligner mapping software (http://bio-bwa.sourceforge.net/) with Maximal Exact Matches (BWA-MEM) alignment algorithm by comparison with the reference genome hg19 [17]. Model-based Analysis of ChIP-Seq (MACS) software (http://liulab.dfci.harvard.edu/MACS/) was used to analyse the ChIP-sequencing data aligned for binding sites prediction. MACS-Peak calling option was applied to retrieve the enriched peak region from ChIP-Seq with input DNA sequence control [18]. 

Motif Analysis of ChIP-Seq Data: Hypergeometric Optimization of Motif EnRichment (HOMER) software (http://homer.ucsd.edu/homer/motif/index.html) was used to identify unknown motifs binding sFRP4 from the ChIP-Seq aligned data. In addition, Gene Cloud software (http://genecloud.org/) was used to predict cPHX1 specific co-functionality genes retrieved from available genomics database.

Gene cluster Analysis: Cluster 3.0 software (http://bonsai.hgc.jp/~mdehoon/software/cluster/) was used to identify interlinking genes of USP9X, FOXK1, and sFRP4 by using functional genomics database.

Generation of heatmap from RNA-Seq data: HeatmapGenerator5 software (https://github.com/Bohdan-Khomtchouk/HeatmapGenerator) was used to describe the quantitative analysis of the gene expression pattern from the whole transcriptome sequencing data [19]. 

### 2.10. sFRP4 RNA Interference

Total RNA from sFRP4-overexpressing U87 cells was isolated using the Trizol method and converted into cDNA using a Verso cDNA Synthesis kit (Invitrogen). Double stranded RNA (dsRNA) was synthesized using MEGAscript^®^ RNAi Kit (Ambion, Austin, TX, USA) as described previously [13]. 

### 2.11. Conversion of WJMSCs into ESC-Like Cells

sFRP4 silenced WJMSCs were cultured in ESC medium containing knockout-DMEM supplemented with 15% knockout serum replacement (KOSR), 2 mM l-glutamine, 0.1 mM non-essential amino acid (NEAA; Gibco, Grand Island, NY, USA), 0.1 mM β-mercaptoethanol, and 4 ng/mL basic fibroblast growth factor (bFGF; BioVison, Milpitas, CA, USA). 

### 2.12. Real-Time Quantitative PCR

Total RNA extraction and cDNA synthesis were carried out using RNeasy Plus Mini kit (Qiagen, Hilden, Germany) and Verso cDNA Synthesis kit (Invitrogen) respectively. Real-time quantitative PCR was performed as described previously [13]. All the primers were purchased from Sigma Aldrich (Bengaluru, India; Table S2). 

### 2.13. Immunocytochemistry

Cells were subjected to treatment and immunocytochemistry was performed as previously described using sFRP4 (Abcam, Cambridge, MA, USA), H2AX (Cell Signaling Technology, Beverly, MA, USA), and β-catenin (BioLegend, San Diego, CA, USA) [11]. 

### 2.14. Western Blotting

Protein expression levels of sFRP4 (Invitrogen), β-catenin (BioLegend), GSK-3β (Cell Signaling Technology), and APAF1 (R&D Systems, Minneapolis, MN, USA) were analyzed by Western blotting as previously described by Warrier et al. [12]. 

### 2.15. Statistical Analysis

Statistical analysis was performed by unpaired Student’s *t*-test and one-way analysis of variance (ANOVA), followed by Dunnett’s post-test analysis and using GraphPad Prism software (v 5.03, GraphPad Software Inc., San Diego, CA, USA). Data are represented as Mean ± SD and all the experiments were done in triplicates. *p* < 0.05 was considered to be statistically significant (* *p* value < 0.05, ^#^ *p* value < 0.01, ^##^ *p* value < 0.001).

## 3. Results

### 3.1. sFRP4 Overexpression has an Anti-Proliferative Effect in Glioma Cell Lines

We observed that in the glioma cell lines, U87MG, U138MG, and U373MG, overexpression of *sFRP4* resulted in inhibition of viability and proliferation as measured by MTT and BrdU assays respectively (Figure S1A,B). Also, apoptosis was increased in sFRP4 OE cells, as measured by ROS and caspase assays (Figure S1C,D), this effect was reversed by knocking down *sFRP4* by specific siRNA (Figure S1A–D). As the cell line U87MG was the most aggressive and rapidly proliferating of all the lines studied, U87MG was used for further studies. 

### 3.2. sFRP4 OE Cells Induces Apoptosis, Inhibits Cancer Stemness

The U87MG cells were transfected with either an empty or an sFRP4 expressing EGFP tagged plasmid, and EGFP expressing cells were confirmed under fluorescent microscopy (Figure S2A). Treatment with sFRP4 siRNA (sFRP4 SI) induced proliferation when compared to control U87 cells, as observed in phase-contrast microscopy (Figure S2B). Apoptotic activity was confirmed by the caspase assay, which showed a significant increase in caspase activity in sFRP4 OE cells. Caspase activity was measured colorimetrically and quantified using flow cytometry (Figure S2C). The inherent cancer stemness property of sphere formation was studied by neurosphere formation assay. The OE cells could not form proper spheres, whereas sFRP4 SI cells formed prominent large spheres compared to untreated control (Figure S2D). Protein levels of sFRP4 increased in sFRP4 OE and was reduced in sFRP4 SI as demonstrated in Figure S2E. Next, gene expression of *sFRP4* was analyzed in sFRP4 OE and sFRP4 SI cells, which confirmed a four- fold increase in *sFRP4* expression in sFRP4 OE and a two-fold decrease in sFRP4 SI cells. In sFRP4 OE cells, the expression of *CycD1*, a cell cycle regulator, was downregulated with the upregulation of pro-apoptotic *Bax*, with a decrease in sFRP4 SI cells but *CycD1* was enhanced in sFRP4 SI cells compared to untreated control (Figure S2F), suggesting that *sFRP4* is correlated with cell cycle progression and apoptosis. These cells were used for further experiments.

### 3.3. sFRP4 Localized in the Nucleus with a DNA Binding Ability

Immunolocalization of sFRP4 in U87 sFRP4 OE cells surprisingly showed sFRP4 nuclear localization, which was not detected in control and SI U87 cells (Figure S3A). The property of nuclear localization was extrapolated by examining the DNA binding property of sFRP4 using DNABIND software. This analysis clearly indicated a DNA binding ability of sFRP4 sequences (Figure S3B).

### 3.4. Senescence Related Genes were Identified in sFRP4 ChIP Sequencing Analysis of sFRP4 OE Cells

**Figure 1:**
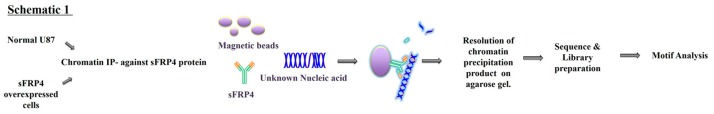

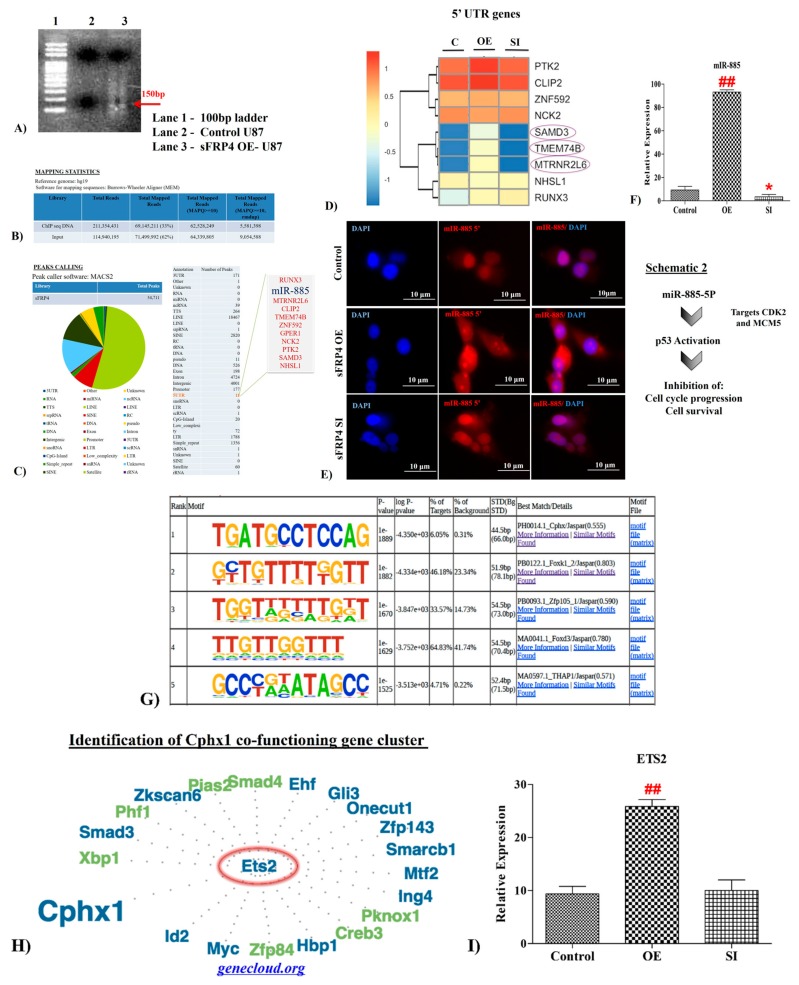
(**A**–**F**). Apoptosis related genes identified in sFRP4 chromatin immunoprecipitation (ChIP) and pull-down sequencing analysis. Schematic 1 represents the sequence of steps of ChIP and downstream sequencing. ChIP DNA resolved on agarose gel indicated a 150 bp DNA band (**A**), ChIP mapping statistics by Burrow-Wheeler Aligner software indicated 5,581,398 mapped reads (**B**); peak calling analysis output using MACS2 software revealed 34,711 peaks related to *secreted frizzled-related protein 4* (*sFRP4*) (**C**, left panel), categorization of peak identities represented by pie chart and table, analysis gene list present within 5′UTR (enlarged) indicated the presence of miRNA 885 (**C**, right panel), RNA sequencing data of 5′UTR revealed an upregulation of three 5′UTR genes in sFRP4 OE (OE) and downregulation in sFRP4 SI (SI) cells as indicated in the box (**D**), upregulation of active miR-885 in sFRP4 OE cells using an miR-885 5′LNA probe was detected as red fluorescence using a fluorescence microscope (scale bar = 10 μm) (**E**), quantitative RT-PCR indicated an over expression of miR885 in sFRP4 OE and downregulation in sFRP4 SI cells (**F**). Schematic 2 represents a model indicating the mode of action of miR-885 through its target genes *CDK2* and *MCM5* in cellular homeostasis via activation of *p53*. Results are mean ± SD of three independent experiments performed in triplicates (* *p* value < 0.05, ^#^ *p* value < 0.01, ^##^ *p* value < 0.001). (**G**–**I**) HOMER de novo motif analysis and gene cluster analysis identifies senescence related genes in sFRP4 OE cells. Table representing LOGOS diagram of first five ranking genes motifs obtained by HOMER motif analysis software (**G**). Gene cluster analysis showing interlinking genes with *Cphx1* gene (first ranked motif) using Gene cloud software identified the senescence associated gene, *ETS2* (**H**). Relative mRNA expression analysis revealed *ETS2* (Cphx1 co-functioning gene) was overexpressed in sFRP4 OE cells (**I**). Results are mean ± SD of three independent experiments performed in triplicates (*p value < 0.05, ^#^ *p* value < 0.01, ^##^ *p* value < 0.001).

When sFRP4 OE cells were subjected to ChIP pull-down with sFRP4 antibody, a 150 bp DNA was observed that was absent in control U87 cells (Figure 1A). The pulled down DNA sample was then subjected to whole-genome ChIP sequencing with input DNA control from U87 cells. 

ChIP sequencing data from sFRP4 OE DNA and input control DNA were mapped by using Burrows-Wheeler Aligner-maximal exact matches (MEMs algorithm) mapping software by comparing with the reference genome HG19. Mapping analysis resulted in total reads of 5,581,398 in sFRP4 OE DNA and 9,054,588 in input DNA (Figure 1B). Peak caller software, MACS2, was used to identify enriched peaks in comparison with the untreated input DNA sequence in which 34,711 enriched genes peaks were identified and categorized based on their chromosomal location (Figure 1C).

#### 3.4.1. UTR Analysis

In the peak-calling annotation, we observed 11 gene peaks at 5’UTR- *RUNX3*, *miR885*, *MTRNR2L6*, *CLIP2*, *TMEM74B*, *ZNF592*, *GPER1*, *NCK2*, *PTK2*, *SAMD3*, and *NHSL1* (Figure 1C). Further, the RNA sequencing data revealed that among all genes, *SAMD3*, *TMEM74B*, and *MTRNR2L6* genes were highly expressed in sFRP4 OE cells but downregulated in sFRP4 SI cells (Figure 1D), of which *SAMD3* (Sterile alpha motif domain-containing protein 3) has been reported to be a cell cycle inhibitor expressed in differentiating cells [20], whereas PTK2 coding for *Focal adhesion kinase* (*FAK*) has a role in apoptosis in human endothelial cells [21]. 

#### 3.4.2. MicroRNA885 Upregulation in sFRP4 OE

Among the UTR peaks identified from the ChIP sequencing data, we observed the presence of *miR885*, which is closely linked to *p53* [22] which we further probed. Interestingly, miR885 was localized in the cytoplasm using 5′ miR885 antisense fluorescent probes by in situ hybridization (Figure 1E). Also, the miR885 expression level was determined by qRT-PCR, and the result indicated that the expression of miR885 in sFRP4 OE cells was four-fold higher than the control U87 cells. miR885 expression was decreased in sFRP4 SI cells (Figure 1F). The role of *miRNA885p* is possibly via activating *p53* by targeting *CDK2* and *MCM5* as previously reported [22]. This possible mode of action of *miR885p* in inhibiting proliferation has been represented in schematic 2.

### 3.5. Motif Analysis

*De novo* motif analysis was done by using HOMER software for sFRP4-ChIP pull-down of sFRP4 OE cells. The top five ranked genes obtained in the analysis represented in the LOGOS diagram were *Cphx1*, *FOXK1*, *Zfp105*, *FOXD3*, and *THAP1* (Figure 1G). *Cphx1* (cytoplasmic polyadenylated homeobox 1), the first gene listed in the motif analysis, is a homeobox gene expressed highly in early embryonic stages [23]. However, a literature search of *Cphx1* suggested that an elaborate functional status of this gene has not yet been studied. Therefore, we examined the co-functional genes of *Cphx1* by using the software genecloud.org, which is a tool to study gene-gene associations based on reported literature (Figure 1H). This analysis revealed that *Cphx1* has a co-functioning gene, the *ETS2* gene, which is a transcription factor regulating genes involved in stem cell development, cell senescence and death, and tumorigenesis [24]. When we examined the expression of this gene in RNA-sequencing data, we found interestingly that *ETS2* was indeed overexpressed in sFRP4 OE cells when compared to control and sFRP4 SI cells (Figure 1I).

### 3.6. Interlinking Analysis of sFRP4 with ChIP Motifs

**Figure 2:**
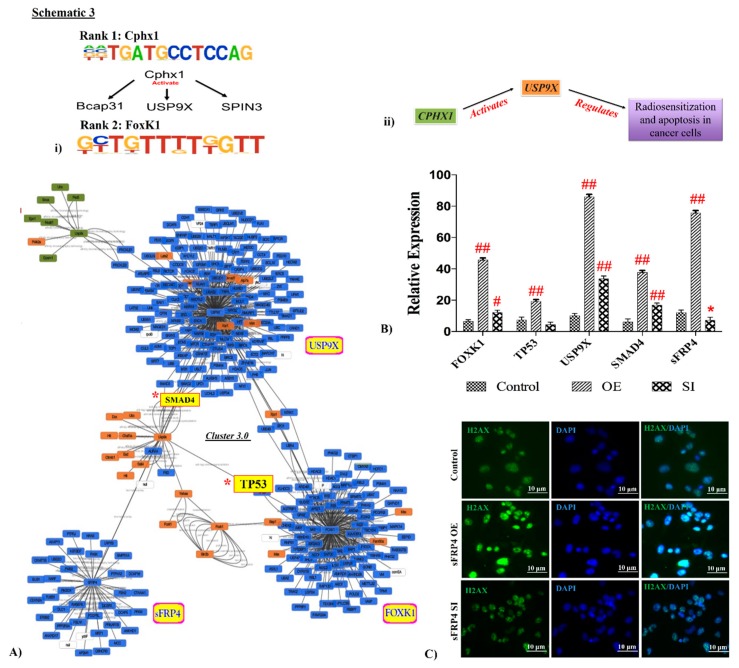
Apoptotic genes *TP53* and *SMAD4* were detected in gene cluster analysis of ChIP genes and interlinking study. Schematic 3 represents LOGOS diagram of first rank motif homeobox-*Cphx1* and second rank motif *FoxK1*, and downstream effectors of *Cphx1* (i) and a flow chart indicating activation of the *Usp9x* gene by *Cphx1* via the reported role of *Usp9x* in the regulation of sensitization and apoptosis in cancer cells (ii). Gene cluster analysis using Cluster 3.0 software indicated linking of *Usp9x*, *Foxk1*, and *sFRP4* via *TP53* and *SMAD4* (**A**). Relative mRNA expression showed upregulation of *FOXK1*, *TP53*, *USP9x*, and *SMAD4* in sFRP4 OE compared to control and sFRP4 SI cells (**B**). DNA instability was indicated in sFRP4 OE cells by immunocytochemical staining of H2AX and visualization using fluorescence microscopy (scale bar = 10 µm) (**C**). Results are mean ± SD of three independent experiments performed in triplicates (* *p* value < 0.05, ^#^ *p* value < 0.01, ^##^ *p* value < 0.001).

The first gene identified in the motif analysis, *Cphx1*, has been reported to activate downstream genes *Bcap31* and *USP9X* during development [23], and therefore we analyzed the expression of these genes in the RNA sequencing data. We observed that *USP9X* gene expression was highly upregulated in sFRP4 OE cells. Further, we used *USP9X* and *FOXK1* (which was ranked second in the motif analysis) and *sFRP4* to identify the gene network interlinking pattern using Cluster 3.0 software. The gene cluster analysis displayed that *SMAD4* and *TP53* genes were interlinked with *sFRP4*, *USP9X*, and *FOXK1* (Figure 2A). RNA sequencing data revealed that expression of *SMAD4* and *TP53* genes was upregulated in sFRP4 OE cells along with *FOXK1* and *USP9X* (Figure 2B). Additionally, p53 pathway activation was confirmed by the upregulation of H2AX protein expression in sFRP4 OE cells using immunofluorescence analysis (Figure 2C), which correlated with earlier reports showing that the H2AX Arf/p53 pathway induces apoptosis in cancer cells [25].

### 3.7. Whole Transcriptome Analysis of sFRP4 OE and sFRP4 SI

Whole transcriptome sequencing was performed to understand the molecular changes occurring during *sFRP4* gene overexpression and silencing. The results have been organized as pathway- and function-specific gene expression patterns. We focused on genes which indicated that the expression was inversely regulated in sFRP4 OE and sFRP4 SI cells, thereby being relevant for *sFRP4*-specific gene activation and suppression.

#### 3.7.1. Cell Cycle Pathway Genes

**Figure 3:**
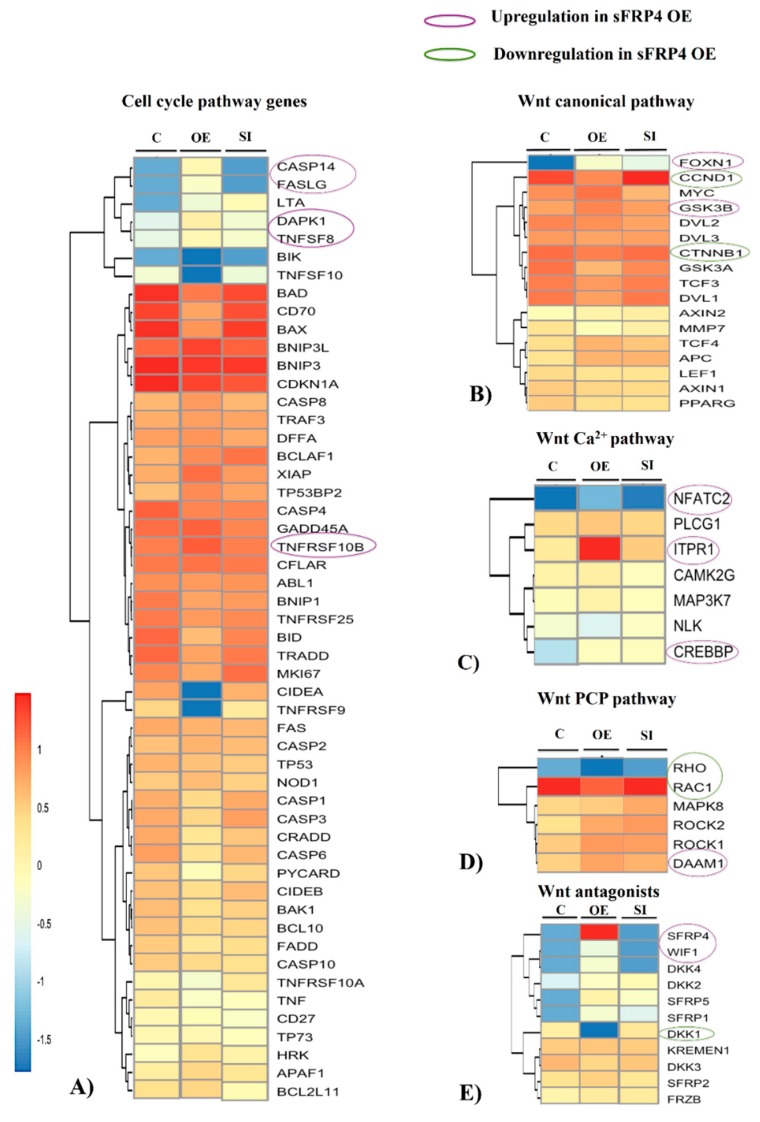

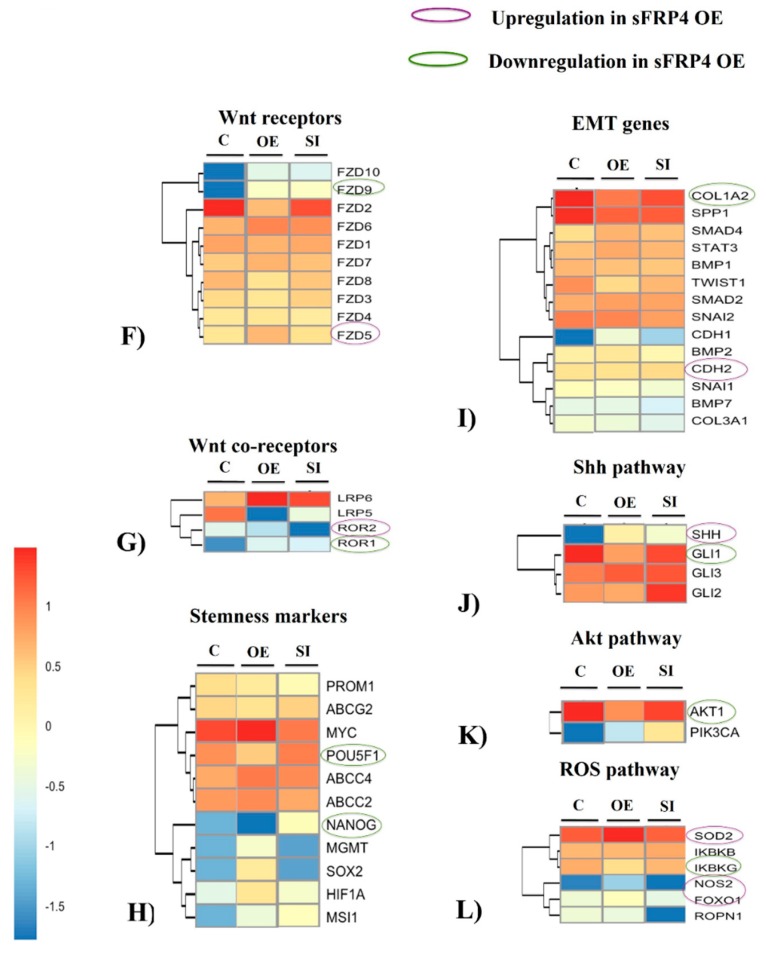
Whole transcriptome analysis of sFRP4 OE, sFRP4 SI, and control indicate variations in expressions between sFRP4 OE and sFRP4 SI. Cell cycle pathway genes (**A**), Wnt canonical pathway (**B**), Wnt Ca^2+^ pathway (**C**), Wnt PCP pathway (**D**), Wnt antagonists (**E**), Wnt receptors (**F**), Wnt co-receptors (**G**), Stemness markers (**H**), EMT genes (**I**), Shh pathway (**J**), AKT pathway (**K**) and ROS genes (**L**). Upregulation indicated by violet circle and downregulation by green circle.

The overall analysis showed a differential gene expression level among treatment conditions. sFRP4 OE cells showed upregulation of *Casp14*, *FASLG*, *DAPK1*, *TNFSF8*, and *TNFRSF10B* genes, which were downregulated in sFRP4 SI cells (Figure 3A). All these gene functions are correlated to cell apoptosis in sFRP4 OE cells, leading to apoptosis in U87 cells. However, the expression pattern of mitochondrial genes *BAX*, *BCL*, and *BAD* was not affected significantly.

#### 3.7.2. Wnt Canonical Pathway

sFRP4 OE cells showed downregulation of *CTNNB1* and *CCND1*, and upregulation of *GSK3β* and *FOXN1* genes. *CTNNB1* (*β-catenin*) and *CCND1* (*Cyclin D1*) are the two central genes of the Wnt canonical pathway maintaining cells’ self-renewal property [26], whereas *GSK3β* and *FOXN1* function as suppressors of *β-catenin* [27,28]. These patterns of gene expression clearly suggested that overexpression of *sFRP4* was inhibiting the Wnt canonical pathway (Figure 3B).

#### 3.7.3. Wnt Non-Canonical Pathways

(a) Wnt calcium pathway:

sFRP4 OE cells displayed an elevation in *ITPR1* gene expression, which encodes for inositol 1,4,5-triphosphate receptor protein activity. The *ITPR1* gene is responsible for maintaining the intracellular IP3-gated calcium channel in Wnt calcium signaling [29,30]. Further, the expression of *NFATC2* and *CREBBP* genes (which are non-canonical pathway-specific transcription factor genes) was upregulated in sFRP4 OE cells, suggesting activation of the Wnt-calcium signaling pathway (Figure 3C).

(b) Wnt-planar cell polarity pathway

The planar cell polarity (PCP) pathway primarily promotes migration of cells with the activation of *RHO* and *RAC* genes, but these genes were shown to be downregulated in sFRP4 OE cells. *DAAM*, another mediator of the PCP pathway, was seen to be upregulated in sFRP4 OE cells (Figure 3D).

#### 3.7.4. Wnt Antagonists

sFRP4 gene expression was increased with complete downregulation of *Dickkopf 1* (*DKK1*) in sFRP4 OE cells (Figure 3E), indicating a possible role of sFRP4 in the regulation of the expression of other Wnt antagonists. In addition to *sFRP4*, *Wnt inhibitory factor 1* (*WIF1*) expression was also increased in sFRP4 OE cells.

#### 3.7.5. Wnt Receptors and Co-Receptors

The frizzled (FZD) family of Wnt receptor genes showed differential regulation among treatments. sFRP4 OE cells exhibited upregulation of *FZD5* and downregulation of *FZD2* when compared to control U87 and sFRP4 SI cells (Figure 3F). The low-density-lipoprotein receptor proteins *LRP5* and *LRP6* were differentially expressed in sFRP4 OE treated cells, with downregulation of *LRP5* and upregulation of *LRP6* (Figure 3G). Although *LRP5* and *LRP6* are co-receptors for the Wnt canonical pathway [31], some reports suggest that *LRP6* may be associated with the Wnt non-canonical pathway, especially in neuronal development [32,33].

#### 3.7.6. Stemness and Epithelial-Mesenchymal Transition (EMT) Markers

Expression of cancer stemness-specific genes *NANOG* and *POU5F1* was inhibited in sFRP4 OE cells, whereas sFRP4 SI cells showed elevation in *NANOG* and *POU5F1* gene expression that was much higher than in the untreated U87 control (Figure 3H). With reference to EMT-specific genes, the mesenchymal marker gene, *COL1A2*, was substantially decreased in sFRP4 OE cells whereas the epithelial specific marker, *CDH1*, was seen to increase in sFRP4 OE cells (Figure 3I).

#### 3.7.7. Sonic Hedgehog (Shh), pI3K-AKT, and ROS Pathways

Expression of *SHH* was upregulated and *GLI1* genes were downregulated in sFRP4 OE cells (Figure 3J). *AKT1* gene expression was downregulated in sFRP4 OE cells, indicating the inhibition of this cell survival pathway (Figure 3K). ROS pathway genes *SOD2*, *NOS2*, and *FOXO1* were upregulated in sFRP4 OE cells, with a decrease of *IKBKG* gene expression (Figure 3L).

### 3.8. Quantification of Wnt Pathway Proteins and Functional Activity

**Figure 4:**
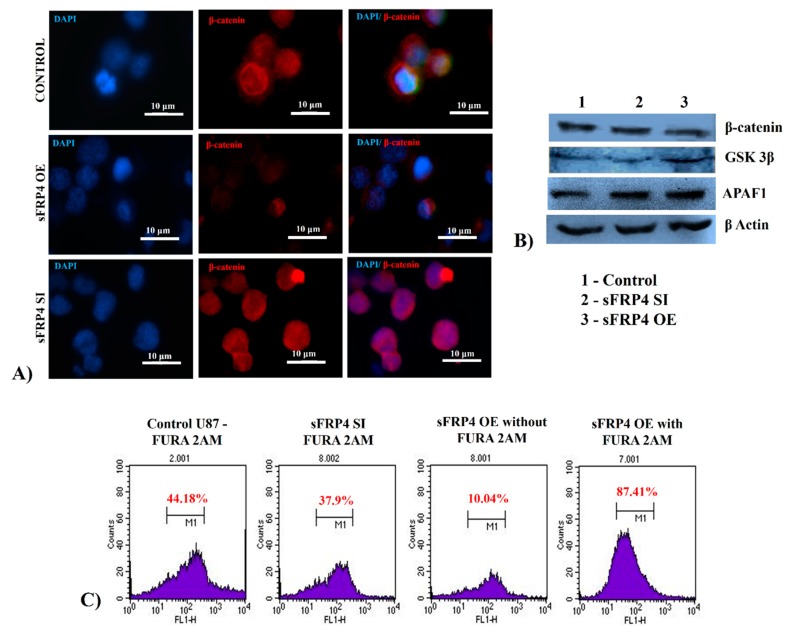
Analysis of intracellular components of Wnt pathway displayed downregulation of Wnt canonical pathway regulators in sFRP4 OE. There was a decrease in β-catenin as shown by immunocytochemical staining of β-catenin (red) in sFRP4 OE cells while there was an increase in nuclear β-catenin in sFRP4 SI cells (scale bar = 10 μm) (**A**). Western blot analysis showed a decrease in β-catenin and increase of GSK3β and APAF1 in sFRP4 OE cells (**B**), FURA-2AM analysis by flow cytometry showed an increase in intracellular calcium level in sFRP4 OE compared to sFRP4 SI cells (**C**).

The β-catenin accumulation in the cytoplasm was decreased in sFRP4 OE cells, whereas in sFRP4 SI cells it was increased when compared to untreated U87 cells (Figure 4A). Western blotting analysis showed a decrease in β-catenin level with an increase of GSK3β level in SFRP4 OE cells. Additionally, upregulation of apoptotic protease activating factor 1 (APAF1), involved in mitochondrial apoptotic pathways, was observed in SFRP4 OE cells confirming the activation of apoptosis in sFRP4 OE cells (Figure 4B). Intracellular Ca^2+^, quantified using flow cytometry, showed a 75–80% increase in calcium levels in sFRP4 OE cells (Figure 4C).

### 3.9. sFRP4 Silencing Increases Pluripotent Property by Activating Wnt Canonical Pathway in Human Wharton’s Jelly Mesenchymal Stem Cells

**Figure 5:**
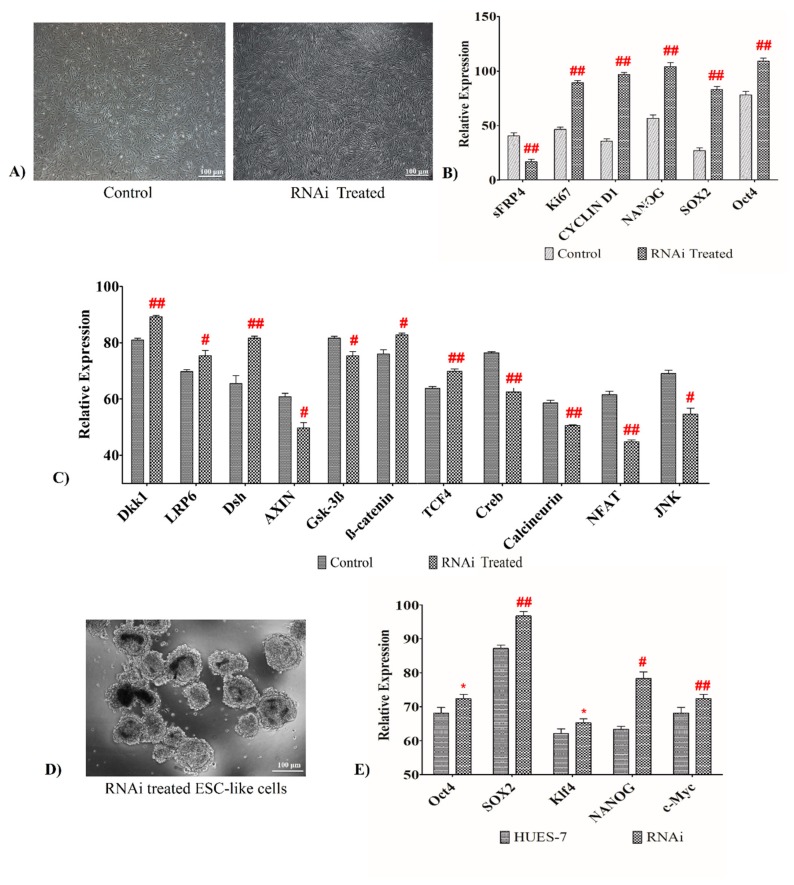
(**A**,**B**). *sFRP4* RNAi treatment on WJ-MSCs displayed upregulation of pluripotent genes and increased proliferation. Photomicrograph images of WJMSCs treated with *sFRP4* RNAi (scale bar = 100 μm) (**A**), Relative mRNA expression of *sFRP4*, *KI67*, *CYCLIN D1*, *NANOG*, *SOX2*, and *OCT 4* in control and *sFRP4* RNAi treated WJMSCs (**B**). Figure 5 (**C**) Gene expression analyses of Wnt specific genes in control and RNAi treated WJMSCs by qRT-PCR. An upregulation of Wnt canonical and downregulation of Wnt non-canonical genes was seen in *sFRP4* RNAi. Figure 5 (**D**,**E**) Conversion of *sFRP4* RNAi-treated WJMSCs into ESC-like cells and upregulation of pluripotent genes. Photomicrograph images of *sFRP4* RNAi-treated WJMSC-derived ESC-like cells (scale bar = 100 μm) (**D**), Gene expression analysis of pluripotent markers for embryonic stem cell line HUES-7 and RNAi-treated WJMSC-derived ESC-like cells showed upregulation of pluripotent markers (**E**). Results are mean ± SD of three independent experiments performed in triplicates (* *p* value < 0.05, ^#^ *p* value < 0.01, ^##^ *p* value < 0.001).

RNA interference (RNAi)-mediated sFRP4 knockdown in human Wharton’s jelly mesenchymal stem cells (WJMSCs) was used to study the effect of *sFRP4*. We observed that *sFRP4* downregulation resulted in excessive proliferation when compared to untreated WJMSCs control (Figure 5A). After *sFRP4* silencing, expression of stemness specific-genes *NANOG* and *OCT4* was upregulated two-folds more than untreated WJMSCs control, and *KI67* and *CYCLIN D1* gene expression was increased significantly in sFRP4 silenced cells (Figure 5B). Further, expression of Wnt canonical genes *β-catenin*, *TCF4*, *LRP6*, *Dsh*, and *Dkk1* increased, whereas *GSK3β* and *AXIN* expression decreased, and that of non-canonical genes *NFAT*, *CalN*, *JNK*, and *CREB* was also seen to decrease in sFRP4 silenced cells (Figure 5C).

### 3.10. sFRP4 Knockdown Elicits Embryonic Stem Cell-Like Traits

To understand the effect of *sFRP4* knockdown on pluripotentiality, *sFRP4* silenced WJMSCs were grown in embryonic stem cells (ESCs) growth medium. This led to the appearance of ESC-like morphology (Figure 5D). Further analysis revealed that these cells express a higher level of genes related to stemness and pluripotentiality, such as *Oct4*, *SOX2*, and *NANOG* as compared to HUES-7 (Figure 5E). This suggests that *sFRP4*-based Wnt antagonism may be involved in the maintenance of stemness of ESCs.

## 4. Discussion

Wnt signaling is one of the key operational pathways regulating embryonic development from the undifferentiated state but is also tightly involved in tumorigenesis. Being an ubiquitous pathway controlling multitude development and disease processes, the Wnt pathway itself is managed by several inducers and inhibitors functioning at various levels of the signaling network. Among the inhibitors of Wnt signaling, the sFRPs are one of the most versatile antagonists of Wnt. We have previously reported that *sFRP4* chemosensitizes glioma stem cells to commonly used chemotherapeutics by decreasing the stemness properties, decreasing drug resistance, and reversing EMT [10,11]. Despite the clear role of *sFRP4* in inhibiting the Wnt pathway and inducing apoptosis via downregulating Wnt effectors, its precise molecular role and targets have not been identified. Therefore, in this study a gain or loss of function of *sFRP4* was attempted in glioma cells to have a deeper understanding of its mechanism of action.

The direct role of *sFRP4* in regulating apoptosis was observed in the *sFRP4* overexpressing and silenced models by virtue of cell death in sFRP4 OE and hyperproliferation in silenced glioma cells. Specific and concurrent epigenetic silencing of the canonical Wnt pathway by Wnt inhibitors, *sFRPs*, *DKKs*, and *WIF* has been shown to increase proliferation in chronic lymphocytic leukemia [34]. However, no specific study has demonstrated that suppressing an extracellular Wnt antagonist can induce proliferation in tumors. The reflection of a significant increase of a pro-proliferative marker *CycD1*, a *β-catenin* effector gene, demonstrated the specificity of the proliferative surge in sFRP4 SI cells. As the gain of expression of *sFRP4* resulted in prominent apoptosis in glioma cell lines, we analyzed whether there was a role of *sFRP4* in apoptosis in the intracellular context and beyond acting via the Wnt pathway.

sFRP4 is a secreted protein predicted to be localized on the cell membrane, intracellularly, and sporadically in the nucleus of various tissues [35]. In our study, we observed that sFRP4 was localized in the nucleus of sFRP4 OE U87 cells but not expressed in control and sFRP4 SI treated U87 cells. The unexpected finding of nuclear localization of sFRP4 was corroborated by DNABIND prediction studies indicating a high probability of DNA interactions. So far there are no reports on the localization of sFRP4 in the nucleus or its affinity to DNA. Based on the structure of sFRPs, it can be hypothesized that the heparin binding sites that have been reported in sFRPs [36,37,38] are involved in the affinity to DNA we observed in this study. In fact, sFRP1was first isolated and identified from the heparin binding fraction of the conditioned medium of fibroblasts from human embryonic lung [36]. Heparin itself mimics the polyanionic structure of DNA, a property which is well exploited commercially [39], and by extension, heparin binding regions in the C-terminal region of sFRP1 [40] could possibly bind DNA.

ChIP sequencing results of sFRP4 OE cells revealed the various genes that could be regulated by *sFRP4*. Peak-calling analysis revealed the presence of 34,711 peaks and they were categorized based on their sequence annotated in the chromosome. Major peaks were on Long Interspersed Element-1 (LINE-1) and non-autonomous Short Interspersed Elements (SINE) regions which are involved in carving the structure and function of mammalian genomes [41,42]. We concentrated on the 5′UTR region involved in transcriptional regulation of genes. Our analysis report showed 11 gene peaks at 5′UTR- *RUNX3*, *miR885*, *MTRNR2L6*, *CLIP2*, *TMEM74B*, *ZNF592*, *GPER1*, *NCK2*, *PTK2*, *SAMD3*, and *NHSL1*, and all their gene activities were upregulated in sFRP4 OE treated cells. Among all genes, *SAMD3*, *TMEM74B*, and *MTRNR2L6* genes were highly expressed in OE cells but downregulated in SI cells. The *SAMD3* gene and its phosphorylated form of protein function as a cell cycle inhibitor and are involved in cell differentiation [43]. The *SAMD3* function is similar to *TGF-β* [44] and *SMAD2* [20] whereas *TMEM* may be involved in autophagosome formation [45]. The *MTRNR2L6* gene coding for humanin-like 6 (HN6), is identified as a neuroprotectant and anti-apoptotic factor [46]. In our study, *SAMD3* and *TMEM74B* genes were upregulated in sFRP4 OE, indicating an activation of apoptosis.

*miR885*, another gene of 5’UTR peaks, has been well studied in neuroblastoma cells, where it was identified to act as a tumor suppressor via TP53-dependent pathway activation leading to cell death [22]. In our study, we have localized miR885 by in situ hybridization using miR885 probes and found that the active form of miR885 was highly expressed in sFRP4 OE U87 cells. Further, the overexpression of *miR885* gene was confirmed in sFRP4 OE cells in comparison to control U87 cells. Therefore, it is possible to hypothesize that the apoptotic process in sFRP4 OE U87 cells could be by *miR885* activation, which is involved in TP53-dependent cell death.

The motif binding analysis from the ChIP sequencing data using homer software indicated *Cphx1*, a homeobox gene implicated during early embryonic development [23], as the first motif. To delve into a possible connection of *Cphx1* in tumorigenesis and senescence, we performed a gene co-function study using the software Gene Cloud, and identified that *Cphx1* co-functions with the *ETS2* gene. *ETS2* is a well-studied transcription factor regulating numerous genes involved in embryonic development, stem cell maintenance, cell senescence, and tumorigenesis [47], and was seen to be overexpressed in OE cells in our study.

To correlate further the significance of *Cphx1*, a previous report indicated that this homeobox gene could function as an activator for *BCAP31* and *USP9X* genes [23]. *BCAP31* did not appear in our RNASeq analysis but *USP9X*, another homeobox gene, was observed to be upregulated in sFRP4 OE U87 cells. By using Cytoscape version 3.4.0 software, we examined the gene network cluster pattern between *USP9X*, *FOXO1* (ranked second in motif site prediction), and *sFRP4* genes, which displayed the interlinking genes such as *SMAD4* and *TP53* genes. Gene expression of *SMAD4* and *TP53* genes was upregulated in sFRP4 OE U87 cells, again indicating apoptotic activation. This to our knowledge is the first study correlating the Wnt antagonist, *sFRP4*, directly to the master apoptotic molecular handle, *p53*.

Our ChIP pull-down analyses indicated that *sFRP4* overexpression in the U87 cells activates numerous apoptosis related events; hence, we pursued a whole transcriptome sequencing to understand sFRP4-specific gene expressional changes. These data revealed an activation of *TNF-death receptor TNFRSF 10B* gene with the upregulation of downstream genes *FASLG* and *caspase 14*. *TP53* activation in cells induces DNA damage and leads to cell death [48]. Owen-Schaub et al., (1995) reported that *TP53* regulates apoptosis by upregulation of the *Fas/APO-1* gene [49]. The *Fas/APO-*1 gene codes for the TNF receptor superfamily member 6 protein, which is known to be a signal inducer for programmed cell death [49]. It supports our finding that sFRP4 OE cells overexpress *TP53* and *FASLG*, which have been correlated with cas14 activation. An activation of the FASLG in connection to Wnt antagonism has not yet been clearly elucidated. The induction of p53-mediated apoptosis via Nutlin-3 activation occurs through overexpression of *FASLG* and initiation of the Fas death receptor pathway in testicular carcinoma [50]. There is a possibility that the intense apoptosis observed upon *sFRP4* overexpression could be mediated by a hitherto unexplored mode of action via the p53- FASLG apoptotic axis.

Expectedly, the classic Wnt canonical pathway was downregulated, as determined by the repression of marker genes *CTNNB1* (*β-catenin*) and *CCND1* (*Cyclin D1*) in sFRP4 OE U87 cells, which was further confirmed by quantification of β-catenin activity by immunolocalization. However, very interestingly, there was a surge in the Wnt calcium pathway with a significant overexpression of the *ITPR1* gene and release of intracellular calcium, as observed in the Fura-2AM assay. Wozniak et al., reported that Fas receptor-mediated apoptosis activates the accumulation of endoplasmic reticulum calcium through upregulation of the *inositol 1,4,5-trisphosphate receptor* (*IP_3_R*) gene [51]. The Fas receptor is involved in activating caspase 8/10 that causes mitochondrial sensitization and releases cytochrome c into the cytoplasm. Cytochrome c binds to *IP_3_R* resulting in intracellular calcium release and mitochondrial calcium overload, leading to cell death [52]. Based on this supporting evidence it is possible to hypothesize that *ITPR1* could induce sFRP4-mediated apoptosis by acting via the Fas receptor-mediated apoptosis. The downregulation of the Wnt PCP pathway genes *Rho* and *Rac* in sFRP4 OE cells suggests an inhibition of the cells’ migratory property by sFRP4.

In addition, the ROS pathway genes *SOD2*, *NOS2*, and *FOXO1* were upregulated in sFRP4 OE cells, which again indicates and supports initiation of apoptosis. An increase in intracellular ROS level causes damage in cell organelles, but cancer cells maintain a high ROS level when compared to normal cells, which helps to maintain CSCs in a limited nutrient environment [53]. However, ROS regulation can be a double-edged sword because excessive ROS accumulation in cells releases more cytochrome c in the cytoplasm and stimulates programmed cell death [54]. Thus, the action of the ROS relies upon its concentration. In our study, the expression of *SOD2* was determined to be two-folds higher in sFRP4 OE cells when compared to control, thereby causing mitochondrial damage. This effect has been supported by a study by Pias et al., which stated that overexpression of SOD induces ROS in mitochondria, leading to apoptosis in PC-21 undifferentiated pheochromocytoma cells [55].

Additionally, the downregulation of the PI3K-AKT pathway genes *AKT1* and *PIK3CA* suggested a dysregulation of this survival pathway. The active state of the PI3K/Akt pathway in cancer has been widely reported in regulating proliferation, metastasis-migration, invasion, and chemo and radioresistance [56,57]. In our study, the expression of survival pathway genes was decreased in sFRP4 OE cells, which indicates the activation of a mechanism promoting apoptosis or growth arrest.

In our studies on the properties of CSCs, the expression of highly specific genes *NANOG* and *POU5F1* were inhibited in sFRP4 OE cells, whereas in sFRP4 SI cells the expression of these genes were elevated more than the untreated control. Furthermore, the expression of drug transporter genes *ABCC2* and *ABCC4* was also upregulated in sFRP4 OE cells. These results indicated that sFRP4 overexpression in U87 cells suppresses cancer chemoresistance via activation of transporter genes. Stemness is closely related to EMT, and in our study, the sFRP4 OE treated cells expressed epithelial marker gene E-cad but the expression of mesenchymal marker gene *COLA1* was significantly decreased. Thus, suppression of mesenchymal gene expression supports the reversal of EMT to mesenchymal-epithelial transition.

sFRP4 SI treatment on U87 cells showed an increase in stem cell gene activation. Therefore, we designed RNAi specific for *sFRP4* to increase the silencing rate. RNAi treatment resulted in a high proliferation rate in U87 cells as compared to untreated U87 cells. The gene expression study revealed the activation of pluripotent genes *NANOG* and *OCT4* with the upregulation of the Wnt canonical pathway specific genes, suggesting that *sFRP4* silencing correlates with stemness increase. These studies point to a direct role of *sFRP4* in suppressing stemness. To further analyze if *sFRP4* is truly involved in regulating stemness and pluripotentiality, we chose to study the effect of *sFRP4* suppression in a non-cancer context. We asked the question if *sFRP4* suppression could convert a multipotent stem cell into a pluripotent state. The data showed that it did indeed and sFRP4 RNAi treatment of multipotent mesenchymal stem cells resulted in a pluripotent stem-like state. An early report on human embryonic germ cell-derived cells has shown the downregulation of *sFRP1* in inducing the proliferation of human ESCs [58]. In Oct4-induced embryonic stem cells, transcriptome analysis revealed that *sFRP1* is downregulated [59]. Our data suggest that knockdown of *sFRP4* alone is sufficient to induce a true pluripotent stem-like phenotype.

## 5. Conclusions

The key findings of this study indicate that sFRP4 is a powerful apoptotic inducer that could act via p53-FASLG axis, and the apoptotic cascade can be fuelled through calcium-based apoptosis acting either through the Wnt calcium pathway or in conjunction with an unknown pathway in which calcium is a key player. Interestingly, the induction of pluripotentiality by sFRP4 knockdown suggests that this antagonist could on its own suppress a highly pluripotent state of stemness.

## Supplementary Materials

The following are available online at http://www.mdpi.com/2072-6694/11/1/25/s1, Figure S1: Viability, proliferation, and apoptosis analysis of glioma cell lines after *sFRP4* overexpression (OE) and silencing (SI), Figure S2: sFRP4 SI initiates proliferation and sFRP4 OE induces apoptotic genes, Figure S3: sFRP4 overexpression (OE) showed functionally active sFRP4 in the nucleus, Table S1: Primers used in siRNA synthesis, Table S2: Primers used in Real-Time PCR.
