# Supplementary material for: Stemness, Pluripotentiality, and Wnt Antagonism: sFRP4, a Wnt antagonist Mediates Pluripotency and Stemness in Glioblastoma
Source: Cancers (Basel). 2018 Dec 27;11(1):25. doi: 10.3390/cancers11010025 (PMC6356444; doi:10.3390/cancers11010025)
Supplement: Supplementary file 1 [file cancers-11-00025-s001.pdf]

Supplementary Materials: Stemness, Pluripotentiality, and Wnt Antagonism: sFRP4, a Wnt antagonist Mediates Pluripotency and Stemness in Glioblastoma

Gurubharathi Bhuvanalakshmi, Naisarg Gamit, Manasi Patil, Frank Arfuso, Gautam Sethi, Arun Dharmarajan, Alan Prem Kumar and Sudha Warrier

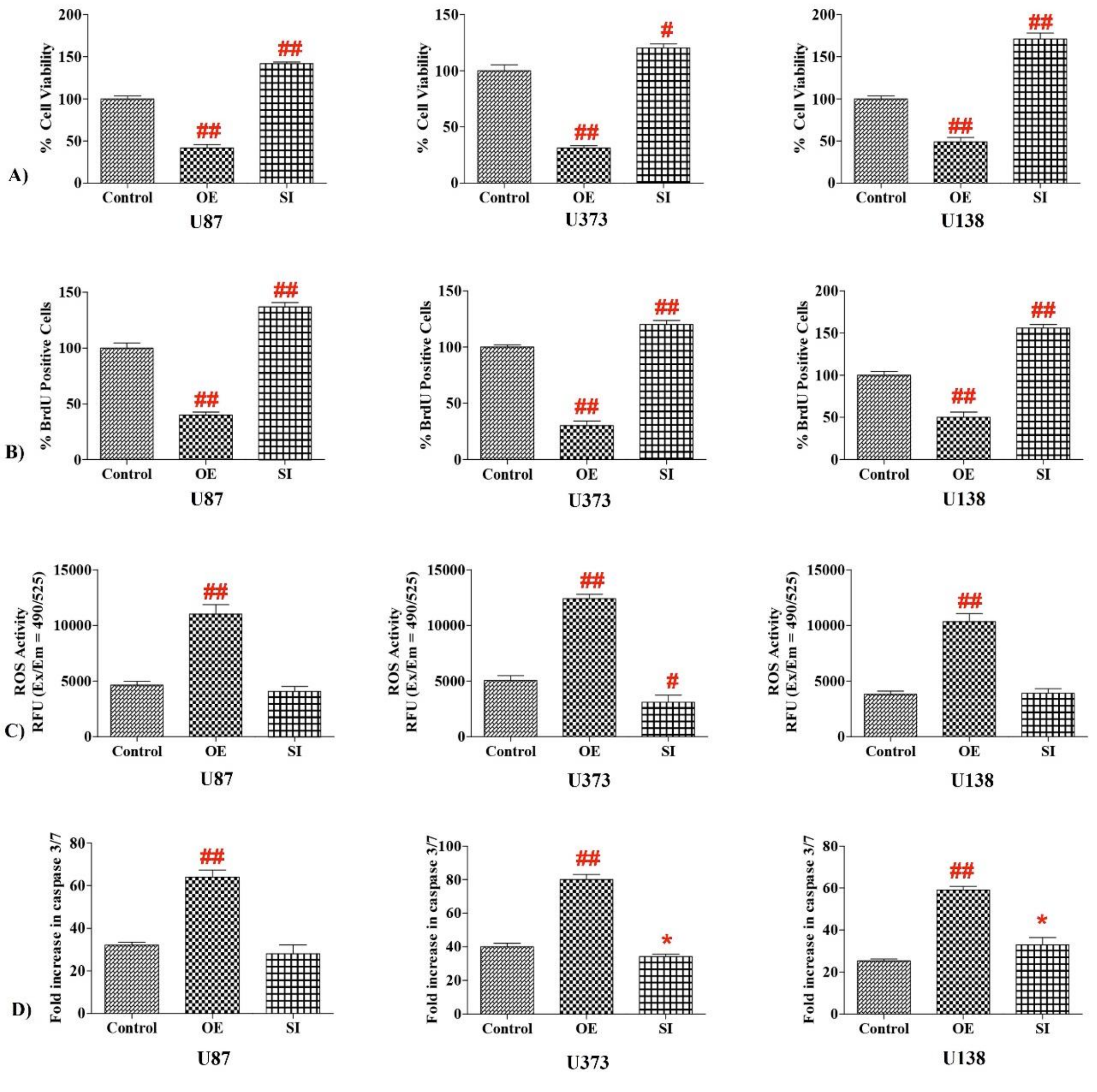

Figure S1. Viability, proliferation, and apoptosis analysis of glioma cell lines after *sFRP4* overexpression (OE) and silencing (SI). U87, U373, and U138 OE and SI cells were analyzed for viability by MTT **(A)** and proliferation by BrdU **(B)** assays, showing inhibition in OE cells and proliferation in SI cells, and an increased release of ROS in OE cells **(C)** and elevated caspase 3/7 activity in OE cells **(D)**. Results are mean ± SD of three independent experiments performed in triplicates (* p value < 0.05, # p value < 0.01, ## p value < 0.001).

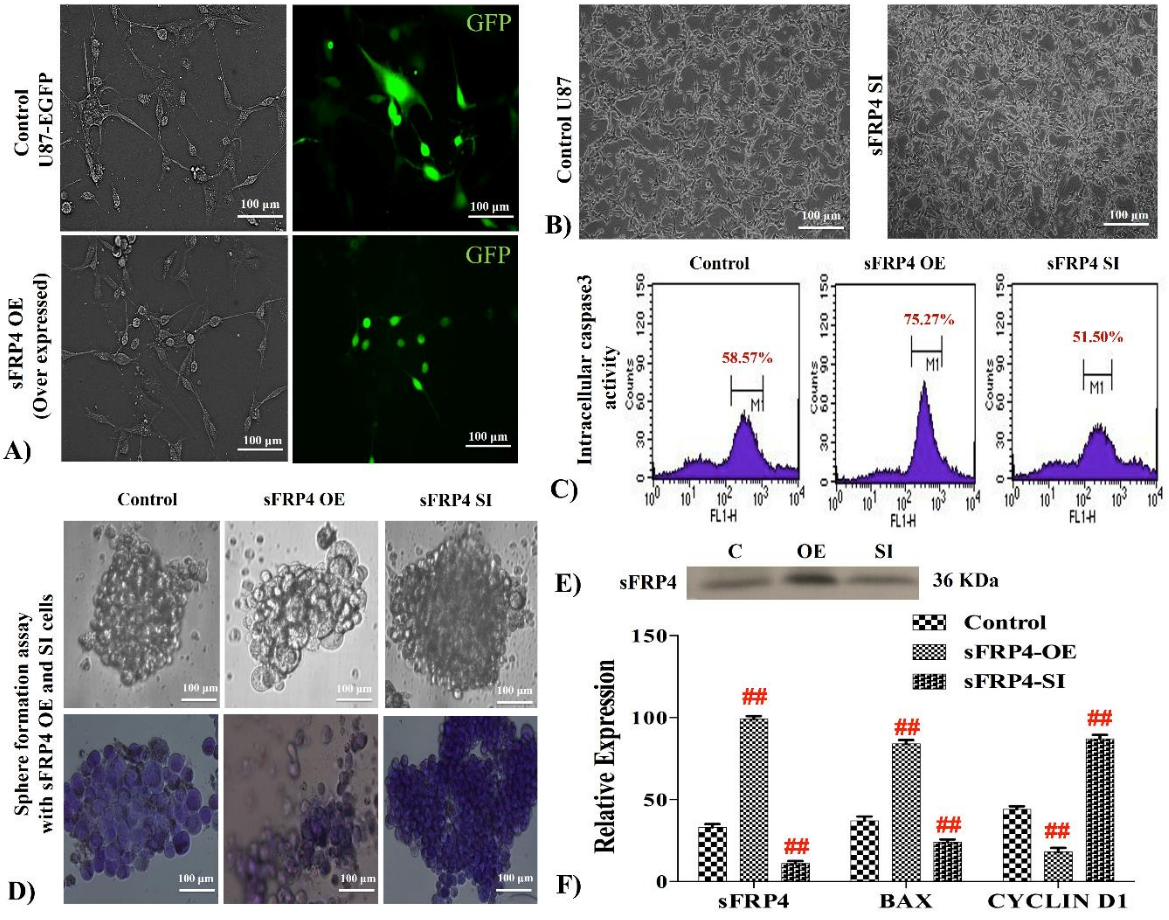

Figure S2. sFRP4 SI initiates proliferation and sFRP4 OE induces apoptotic genes. Photomicrograph images of control and sFRP4-GFP overexpressing U87 cells analyzed by phase contrast microscopy (left) and GFP labeling analyzed by fluorescence microscopy (right) (**A**), photomicrographs showing proliferation of U87 cells treated with sFRP4 SI as compared to control (scale bar = 100 µm) (**B**), an increase in intracellular caspase in sFRP4 OE cells was determined by flow cytometry (**C**), disruption in neurospheres analyzed by sphere formation ability of U87 cells in OE and SI conditions as shown by phase contrast images (top panel) and stained with crystal violet (bottom panel) (**D**), western blot analysis showed an increase in sFRP4 protein in sFRP4 OE and decrease in sFRP4 SI (**E**), representative graphs showing relative mRNA expression of *sFRP4*, *BAX*, and *CYCLIN D1* in sFRP4 OE and sFRP4 SI treated cells (**F**). Results are mean ± SD of three independent experiments performed in triplicates (* p value < 0.05, # p value < 0.01, ## p value < 0.001).

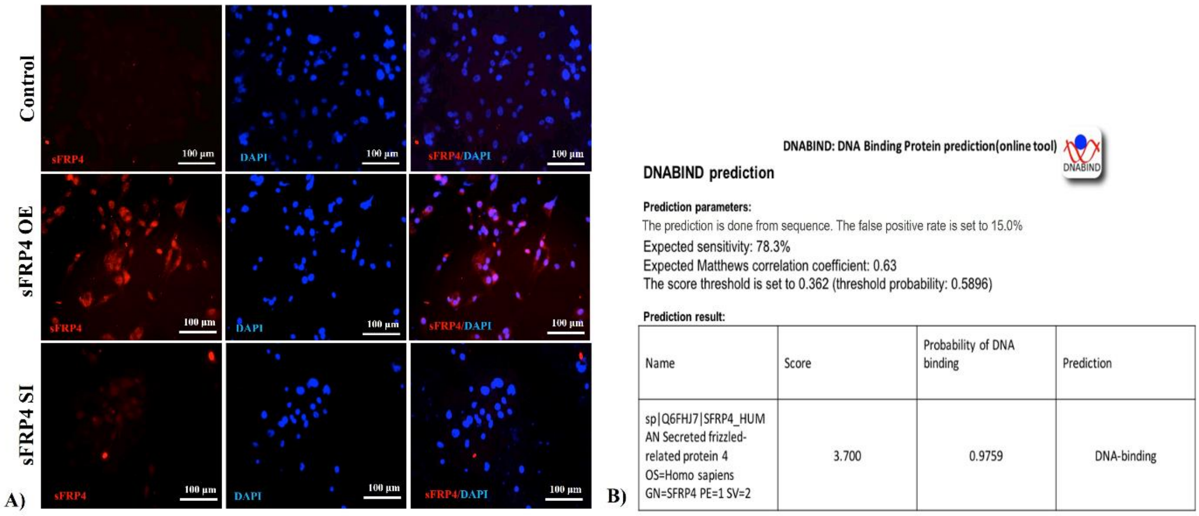

Figure S3. sFRP4 overexpression (OE) showed functionally active sFRP4 in the nucleus**.** The presence of sFRP4 in the nucleus of sFRP4 OE cells was determined by immunocytochemistry (scale bar = 100 µm) (**A**) and by DNA binding prediction by DNABIND prediction tool (**B**).

Table S1 Primers used in siRNA synthesis.

siRNA Name	Primer Sequence
sFRP4 CDS target	5′ AAGTCCCGCTCATTACAAATT 3′
siRNA Sense	5′ AAGUCCCGCUCAUUACAAAUU 3′
siRNA Anti-Sense	5′ AAUUUGUAAUGAGCGGGACUU 3′
siRNA Scrambled Sense	5′ GACAGTACCTATCATCCTATA 3′
siRNA Scrambled Anti-Sense	5′ TATAGGATGATAGGTACTGTC 3′

Table S2 Primers used in Real-Time PCR.

Genes	Primer Sequence	Base Pair	Annealing Temperature (°C)
sFRP4<br>(NM_003014.4)	F:5′ CGATCGGTGCAAGTGTAAAA 3′<br>R:5′ GACTTGAGTTCGAGGGATGG 3′	181	54
BAX<br>(NM_138761.4)	F: 5′ GCTGGACATTGGACTTCCTC 3′<br>R: 5′ TCAGCCCATCTTCTTCCAGA 3′	167	61
CYCLIN D1<br>(NM_053056.2)	F: 5′ AACTACCTGGACCGCTTCCT 3′<br>R: 5′ CCACTTGAGCTTGTTCACCA 3′	204	61
NANOG<br>(NM_024865.4)	F: 5′ TTTGTGGGCCTGAAGAAAACT 3′<br>R: 5′ AGGGCTGTCCTGAATAAGCAG 3′	116	55
SOX2<br>(NM_003106.4)	F: 5′ TCAGGAGTTGTCAAGGCAGAG 3′<br>R: 5′ TCCGGGCTGTTTTTCTGGTT 3′	520	60
OCT4<br>(NM_002701.6)	F: 5′ AGGGCAAGCGATCAAGCA 3′<br>R: 5′ GGAAAGGGACCGAGGAGTA 3′	168	60
Klf4<br>(NM_004235.6)	F: 5′ GCAGTTTCCCGACCAGAGAG 3′<br>R: 5′ GCGAGTAAGTAGGTCCGGTG 3′	370	53.5
c-Myc<br>(NM_002467.5)	F: 5′ GCGTCCTGGGAAGGGAGATCCGGAGC 3′<br>R: 5′ TTGAGGGGCATCGTCGCGGGAGGCTG 3′	328	65
Ki67<br>(NM_002417.5)	F: 5′ TCCTTTGGTGGGCACCTAAGACCTG 3′<br>R: 5′ TGATGGTTGAGGTCGTTCCTTGATG 3′	156	55
β-catenin<br>(NM_030877.5)	F: 5′ CGTCCACAACACTCTGGCTA 3′<br>R: 5′ GCCAGCACTTCACTGCAATA 3′	159	55
Dkk1<br>(NM_012242.4)	F: 5′ TCCGAGGAGAAATTGAGGAA 3′<br>R: 5′ CCTGAGGCACAGTCTGATGA 3′	157	52
GSK3β	F: 5′ ACTCCAGTGGCGAGAAGAAA 3′	241	58

(XM_006713610.3)	R: 5′ TTGAGGACAGCAGTGTCAGG 3′		
LRP6<br>(NM_002336.3)	F: 5′ AGGCACTTACTTCCCTGCAA 3′<br>R: 5′ GGGCACAGGTTCTGAATCAT 3′	274	54
AXIN<br>(NM_003502.4)	F: 5′ CGAGAGCCATCTACCGAAAG 3′<br>R: 5′ TTTTCCTCCATAGTGGCCTG 3′	166	54
TCF4<br>(NM_001146274.2)	F: 5′ CGTAGACCCCAAAACAGGAA 3′<br>R: 5′ TCCTGTCCTTGATTGGGTACA 3′	155	53
CREB<br>(NM_004379.5)	F: 5′ ATGACCATGGAATCTGGAGC 3′<br>R: 5′ GGGCTAATGTGGCAATCTGT 3′	112	58
NFAT<br>(XM_017025783.2)	F: 5′ TTCGGAAAGGAGAGACGGAC 3′<br>R: 5′ ACAGGACCATCTTCTTCCCG 3′	213	55
Calcineurin<br>(NM_000945.4)	F: 5′ GATGATGGTGGGGAACAATC 3′<br>R: 5′ GCCACCTACAACAGCACAGC 3′	127	58
JNK1<br>(XM_024448080.1)	F: 5′AGAAGCTCCACCACCAAAGA 3′<br>R: 5′CTGTGCTAAAGGAGAGGGCT 3′	154	55
Dsh<br>(NM_004423.4)	F: 5′ TCACCCTGACTGTAGCCAAG 3′<br>R: 5′ AGGGATGGAACTGGTGATGG 3′	203	56
GAPDH<br>(NM_002046.7)	F: 5′ CAGAACATCATCCCTGCATCCACT 3′<br>R: 5′ GTTGCTGTTGAAGTCACAGGAGAC 3′	258	61

© 2018 by the authors. Licensee MDPI, Basel, Switzerland. This article is an open access article distributed under the terms and conditions of the Creative Commons Attribution (CC BY) license (http://creativecommons.org/licenses/by/4.0/).
